# The *Acinetobacter* trimeric autotransporter adhesin Ata controls key virulence traits of *Acinetobacter baumannii*

**DOI:** 10.1080/21505594.2018.1558693

**Published:** 2019-01-14

**Authors:** Marko Weidensdorfer, Masahito Ishikawa, Katsutoshi Hori, Dirk Linke, Bardya Djahanschiri, Ruben Iruegas, Ingo Ebersberger, Sara Riedel-Christ, Giulia Enders, Laura Leukert, Peter Kraiczy, Florian Rothweiler, Jindrich Cinatl, Jürgen Berger, Katharina Hipp, Volkhard A. J. Kempf, Stephan Göttig

**Affiliations:** a Institute for Medical Microbiology and Infection Control, University Hospital, Goethe University, Frankfurt, Germany; b Department of Biotechnology, Graduate School of Engineering, Nagoya University, Nagoya, Japan; c Department of Biosciences, Section for Genetics and Evolutionary Biology, University of Oslo, Oslo, Norway; d Department for Applied Bioinformatics, Institute of Cell Biology and Neuroscience, Goethe University, Frankfurt, Germany; e Senckenberg Biodiversity and Climate Research Centre Frankfurt (BIK-F), Frankfurt, Germany; f Institute of Medical Virology, University Hospital, Goethe University, Frankfurt, Germany; g Electron Microscopy Facility, Max Planck Institute for Developmental Biology, Tübingen, Germany

**Keywords:** Adhesion, endothelial cells, host cell response, HUVEC, *Galleria mellonella*

## Abstract

*Acinetobacter baumannii* is a Gram-negative pathogen that causes a multitude of nosocomial infections. The *Acinetobacter* trimeric autotransporter adhesin (Ata) belongs to the superfamily of trimeric autotransporter adhesins which are important virulence factors in many Gram-negative species. Phylogenetic profiling revealed that *ata* is present in 78% of all sequenced *A. baumannii* isolates but only in 2% of the closely related species *A. calcoaceticus* and *A. pittii*. Employing a markerless *ata* deletion mutant of *A. baumannii* ATCC 19606 we show that adhesion to and invasion into human endothelial and epithelial cells depend on Ata. Infection of primary human umbilical cord vein endothelial cells (HUVECs) with *A. baumannii* led to the secretion of interleukin (IL)-6 and IL-8 in a time- and Ata-dependent manner. Furthermore, infection of HUVECs by WT *A. baumannii* was associated with higher rates of apoptosis via activation of caspases-3 and caspase-7, but not necrosis, in comparison to ∆*ata*. Ata deletion mutants were furthermore attenuated in their ability to kill larvae of *Galleria mellonella* and to survive in larvae when injected at sublethal doses. This indicates that Ata is an important multifunctional virulence factor in *A. baumannii* that mediates adhesion and invasion, induces apoptosis and contributes to pathogenicity *in vivo*.

## Introduction

The Gram-negative γ-proteobacterium *Acinetobacter baumannii* is an important pathogen in the hospital environment and causes a multitude of nosocomial infections including wound and urinary tract infections, pneumonia and bloodstream infections. The ability to acquire or upregulate antimicrobial resistance determinants has led to a high frequency of multidrug-resistant *A. baumannii* strains worldwide [1,2]. In 2017, the World Health Organization classified carbapenem-resistant *A. baumannii* as “priority one” on the global priority list of antibiotic-resistant, pathogenic bacteria for research and development of new antibiotics [3].

To establish infections, pathogenic bacteria need to adhere to human host cells and tissues which is often followed by bacterial invasion. Among others, biofilm formation, modulation of host cell signaling, induction of apoptosis, serum resistance and immune evasion support to maintain an infection. Several proteins of *A. baumannii* have been described to be involved in this processes: e.g. the outer membrane protein A (OmpA) mediates adhesion to epithelial cells and cytotoxicity [4,5], phospholipases D (PLD) support invasion and pathogenicity *in vivo* [6], and the plasminogen-binding protein (CipA) inactivates the alternative complement system and supports the penetration of endothelial cell layers [7]. Previously, the trimeric autotransporter adhesin Ata, was identified in *A. baumannii* ATCC 17978, which mediated adhesion to extracellular matrix proteins (ECMs) and virulence in a murine pneumonia model [8,9]. Trimeric autotransporter adhesins (TAAs) are important virulence factors in Gram-negative bacteria, that regulate adhesion, biofilm formation, immune evasion, angiogenesis or cell death [10–13]. *In silico* analyzes from sequenced *Acinetobacter* spp. strains revealed that species harbouring *ata* in their genomes span the full diversity of the genus *Acinetobacter* [14]. In *A. baumannii* ATCC 19606, the domain architecture of Ata comprises a duplicated head domain, a repetitive neck-stalk region, and membrane anchor domains (Supp. Figure 1) [14].

**Figure 1.**
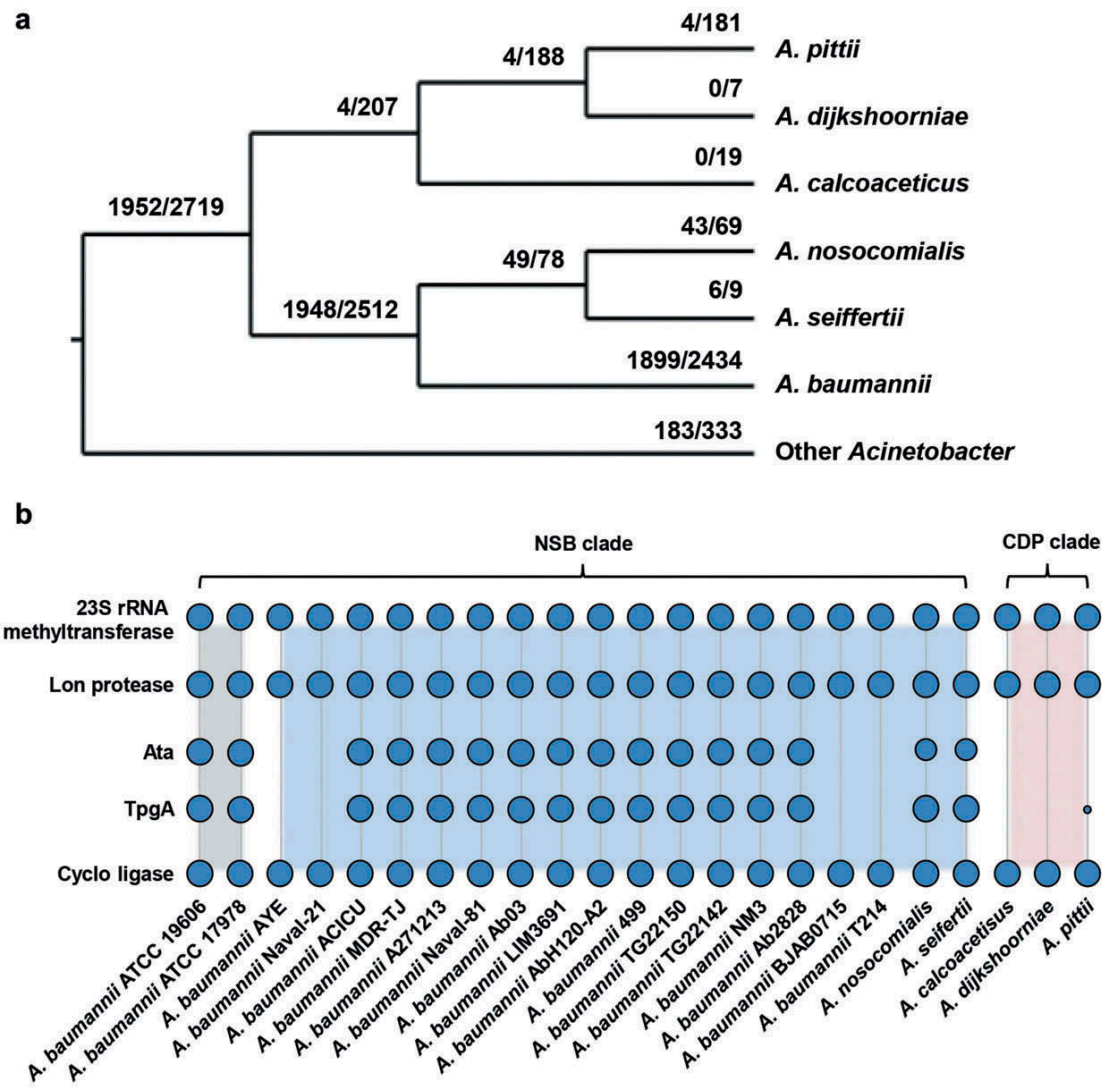
The phylogenetic distribution of *ata*. (a) The tree displays the prevalence of *ata* in individual *Acinetobacter* spp.. Branch labels denote the number of genomes harboring *ata* vs. the total number of analyzed genomes in the corresponding clade. Species outside the *Acinetobacter calcoaceticus-baumannii* complex are collapsed into a single taxon named “Other *Acinetobacter”*. The tree topology follows Poppel et al. [62]. (b) Phylogenetic profile of the *A. baumannii* ATCC 19606 gene cluster encoding the following five proteins: 23S rRNA methyltransferase – Lon protease – Ata – TpgA – Cyclo-ligase. Blue dots indicate the presence of a gene’s ortholog in the respective taxon. Dot sizes are proportional to the fraction of genomes subsumed in each taxon harboring an ortholog. The presence/absence information is given per strain in the case of *A. baumannii* and is summarized on the species level for the other species (see Figure 5(a) for the number of analyzed genomes). The profiles of the two reference strains are shaded in grey. Further 16 profiles of *A. baumannii* strains exemplifying the variation within this species are depicted in the blue shaded area together with profiles of the two further species in the NSB clade. The profiles for the CDP clade are shaded in red. Ata and TpgA orthologs are absent in almost all analyzed genomes in this clade, while the remaining three genes are consistently present.

Recently, we could demonstrate that Ata mediates adhesion to human primary endothelial cells under static and shear-stress conditions *in vitro* and in an *ex vivo* human organ infection model [15]. The function of Ata during *A. baumannii* infections is still poorly understood and nothing is known about the host cell-interacting ligands of *A. baumannii*. Former studies investigated *Acinetobacter* virulence using epithelial cells as host targets [16,17], but it can be assumed that different cell types show different host-pathogen interaction patterns. We therefore aimed to characterize and compare the role of Ata in host-pathogen interaction by analyzing adhesion, invasion, host-cell-modulation and apoptosis in endothelial and epithelial cells.

## Materials and methods

### Computational analysis

For the evolutionary characterization of Ata the encoding sequence at position 315,815 to 322,591 in the genome of *A. baumannii* ATCC 19606 (NCBI Reference Sequence: NZ_KL810966) was used. We determined the phylogenetic profile of *ata*, i.e. the presence-absence pattern of its orthologs across 3052 *Acinetobacter* spp. genomes available in the NCBI RefSeq data base version 87, representing 58 *Acinetobacter* species. A two-step procedure was implemented to reduce the computational complexity of the analysis: As the first step, cliques of orthologous proteins across a core set of 103 *Acinetobacter* spp., encompassing pathogenic strains as well as type and reference species (NCBI RefSeq assembly and protein accessions listed in **Supp. List**), were compiled using OMA standalone 2.0.1 [18] with default parameters. The resulting core orthologous groups were then extended with sequences from the remaining 2949 *Acinetobacter* spp. genomes using the targeted ortholog search tool HaMStR v.13.2.9 (https://github.com/BIONF/hamstr; [19]). HaMStR was run with the parameters ‘-central -force -checkCoorthologsRef -scoreThreshold -filter = F‘. From the resulting extended orthologous groups, we finally extracted and visualized the phylogenetic profiles using PhyloProfile [20]. Domain annotations of the encoded proteins were determined by daTAA tool [21].

### Generation of parkm expression vector and transconjugation

An isogenic knock-out strain of *ata* (∆*ata*) was previously generated in *A. baumannii* ATCC 19606 [15]. For complementation of ∆*ata*, the pARP3 plasmid [22] was digested with *Pvu*II to remove the ampicillin resistance marker. Plasmids and oligonucleotides used for cloning are listed in Table 1 and **Supp. Table 1.** The kanamycin resistance gene cassette was amplified from pET42a using Kan_*Pvu*II_fwd/Kan_*Pvu*II_rev, digested with *Pvu*II, and cloned into the *Pvu*II site of pARP3, generating pARKM. *ata* and its SD sequence were amplified from *A. baumannii* ATCC 19606 using *ata*-in_fusion-fwd/*ata*-in_fusion-rev and cloned into the *Eco*RI-*Xba*I site of pARKM by In-fusion PCR cloning (Clontech), generating pARKM_*ata*. The resulting plasmids were transconjugated from *Escherichia coli* WM6026 (Table 1) [23] into ∆*ata*. For this, ∆*ata* was grown in lysogeny broth (LB), whereas *E. coli* WM6026 harboring pARKM or pARKM_*ata* was grown overnight in LB containing 50 µg/mL kanamycin and 600 µM diaminopimelic acid (DAP). Equal volumes of bacterial suspensions were mixed and spotted on LB agar containing 600 μM DAP. Samples were incubated for 24 h at 37°C and plated onto LB agar plates containing 100 µg/mL of kanamycin for selection of positive transformants: ∆*ata*::pARKM, termed ∆*ata*_(p) (empty vector control) and ∆*ata:*:pARKM_*ata*, termed ∆*ata*_(c) (complemented strain).

**Table 1.** Bacterial strains and plasmids.

	Designation	Characteristic/Comments	Reference
Bacterial Strains	*A. baumannii* ATCC 19606	Type strain, isolated from humans, expressing *ata*	GenBank accession no. ACQB00000000.1
	*A. baumannii* ATCC 19606 ∆*ata*	*ata* markerless deleted mutant (positions 315,815–322,591)	[15]
	*A. baumannii* ATCC 19606 ∆*ata*::pARKM [Δ*ata_*(p)]	*A. baumannii* ATCC 19606 ∆*ata* transformed with pARKM, Kan^R^, Gen^R^, pBAD-Promotor	this study
	*A. baumannii* ATCC 19606 ∆*ata:*:pARKM_*ata* [Δ*ata_*(c)]	*ata* gene cloned into pARKM, transformation of *A. baumannii* ATCC 19606 ∆*ata* for complementation	this study
	*A. baumannii* ATCC 17978	Reference strain, isolated from humans, expressing *ata*	Gottfried Wilharm, Wernigerode
	*A. baumannii* ATCC 17978 Δ*ata*	*ata* transposon mutant, transformed with EZTn5 <KAN-2> (Epicentre Biotechnologies, Madison, Wisconsin, USA)	Gottfried Wilharm, Wernigerode
	*E. coli* DH5α	Host strain used for cloning	New England Biolabs
	*E. coli* BL21 (DE3)	Host strain for protein production	New England Biolabs
	*E. coli* WM6026	Donor strain for bacterial conjugation to *A. baumannii* ATCC 19606 ∆*ata*	[23]
Plasmids	pARP3	Shuttle vector for *E. coli-A baumannii* and expression vector under pBAD-promotor for *A. baumannii*, Amp^R^, Gen^R^	[22]
	pARKM	Shuttle vector for *E. coli-A. baumannii* and expression vector under pBAD-promotor for *A. baumannii*, Kan^R^, Gen^R^	this study
	pARKM_*ata*	Full length *ata*-gene incl. SD sequence cloned into pARKM for expression of *ata*, Kan^R^, Gen^R^	this study
	pTOPO	Cloning vector for amplicons with blunt ends, *lac*Zα for blue/white screening, Kan^R^, Zeo^R^	Thermo Fisher Scientific
	pTOPO_TA_2.1_*hmbs*	Partial sequence of *hmbs* cloned into pTOPO_TA.2.1, external standard for qRT-PCR, Kan^R^, Amp^R^	[15]
	pTOPO_TA_2.1_*rpoB*	Partial sequence of *rpoB* cloned into pTOPO_TA_2.1, external standard for qRT-PCR, Kan^R^, Amp^R^	[15]
	pTOPO_*ata*	Partial sequence of *ata* cloned into pTOPO_TA_2.1, external standard for qRT-PCR, Kan^R^, Zeo^R^	this study
	pET24a	Expression vector for *E. coli* under regulation of *lac*-promotor, Kan^R^	Novagen
	pET24a_ata-head	Sequence of *ata* head domain cloned into multiple cloning site of pET24a, Kan^R^	this study

### Cultivation of human cells and bacterial strains

Primary human umbilical cord vein cells (HUVECs) were prepared from fresh cord veins according to the ethical permission (4/12; University Hospital Frankfurt am Main) as described [24]. Human endothelial cells (HMEC-1 and HDMEC), epithelial cell lines (A549, HeLa and HepG-2) and the monocytic cell line THP-1 were purchased from either Promocell (Heidelberg, Germany) or DSMZ (Braunschweig, Germany) and cultivated as described in **Suppl. Methods**. Bacteria used in this study are listed in Table 1. Cultivation conditions and preparation of bacteria for infection experiments are shown in **Suppl. Methods**.

### Generation of an anti-ata antibody

A polyclonal anti-Ata antibody targeted against the head domain of Ata was generated by immunization of rabbits with a recombinant protein (~68 kDa, monomeric) which was purified from *E. coli* BL21 (DE3) transformed with pET24a_*ata*-head (Table 1). Details are given in Supp. Table 1 and in **Supp. Methods**.

### Analysis of *ata* gene expression and ata protein production

Quantification of *ata* gene expression was done as described in [25] and **Supp. Methods** by RNA isolation and subsequent quantitative real-time PCR (qRT-PCR) amplifying *rpoB* and *ata* using primers listed in Table 1. Immunostaining of Ata and fluorescence microscopy was conducted as described in **Supp. Methods**.

### Analysis of bacterial adhesion to human cells and invasion

Adhesion of *A. baumannii* to human cells was analyzed as described previously [15]. Visualization of bacterial adhesion to HUVECs was done by fluorescence microscopy and by scanning electron microscopy (see **Supp. Methods** for technical details). Invasion of *A. baumannii* into host cells was carried out by a gentamicin/colistin-protection assay. Human cells were seeded into 6-well plates and infected with *A. baumannii* (MOI 200) for 4 h. Subsequently, samples were washed three times with the respective cell growth medium and 1 ml of gentamicin-sulfate (500 µg/mL, WT and *∆ata*) or colistin (10 µg/mL, *∆ata_(p)* and *∆ata_(c)* harboring a gentamicin resistant cassette) was added to the infected cells to kill extracellular bacteria. After 2 h of incubation, cells were washed and lysed by addition of 1 mL *A. dest*.. Afterwards, serial dilutions were plated onto LB agar for CFU determination.

### Analysis of cell death and caspase activity

HUVECs were seeded into 6-well plates (5 × 10^5^ cells) and infected with *A. baumannii* (MOI 1) or incubated with 60 µM camptothecin (CAT, positive control) for 8, 16 and 24 h. Thereafter, cells were washed and detached from the wells using a cell scraper. Endothelial cells were stained with Annexin V-FITC and propidium iodide (BD Bioscience) and induction of apoptosis or necrosis was monitored by flow cytometry (BD Bioscience). Activity of caspase-3 and caspase-7 was determined in 96-well plates with 1 × 10^4^ cells infected with *A. baumannii* (MOI 1) using the Caspase-Glo® 3/7 Assay (Promega) according to manufacturer’s instructions. Cytotoxicity of *A. baumannii* towards HUVECs was measured by a lactate dehydrogenase (LDH) assay (**Supp. Methods**).

### *Galleria mellonella* infection model

*In vivo* pathogenicity was analyzed in larvae of *G. mellonella* as described previously [26]. Survival of *A. baumannii* in *G. mellonella* larvae was investigated by injecting 1 × 10^5^ bacteria into the last left proleg, followed by incubation at 37°C. Immediately after injection and after 24, 48 and 72 h, larvae were homogenized and serial dilutions were plated onto Endo agar (Oxoid) for CFU determination. For quantification of hemocytes in *G. mellonella*, larvae were infected as described above and incubated for 72 h. For the indicated time points, larvae were cut with a scalpel and centrifuged to separate the hemolymph. Hemocytes were resuspended in trypsin-EDTA (0.05%, Gibco), stained with trypan blue and enumerated using a hemocytometer.

### Quantification of cytokines and transmigration of THP-1 cells

Quantification of cytokines and chemokines in the supernatants of infected HUVECs was done employing an enzyme-linked immunosorbent assay (ELISA) (Microbial-induced Multi-Analyte ELISArray Kit, Qiagen) or by the human IL-6 or IL-8 ELISA kit (BD Bioscience) (**Supp. Methods**). Transmigration of monocytic THP-1 cells to infected HUVECs was evaluated as described [27] and in **Supp. Methods**.

### Statistical analysis

Experiments were performed at least three times and differences between mean values of experimental and control groups were analyzed by Student’s t-test (Graph Pad Prism 5.0; Graph Pad Software, San Diego, CA, USA). A p-value of p < 0.05 was considered to be statistical significant. Median lethal doses (LD_50_) were calculated by non-linear regression analysis as described [26].

## Results

### Prevalence of *ata* within the genus *acinetobacter*

We performed an *in silico* screen for the presence of *ata* in 3,052 *Acinetobacter spp*. genomes. This revealed orthologs in 2,135 genomes covering the full phylogenetic diversity of this genus. This indicates that *ata* was already present in the last common ancestor of this genus. However, the prevalence of *ata* in the individual species varies considerably (Figure 1(a)). In case of the monophyletic *A. nosocomialis, A. seifertii*, and *A. baumannii* (NSB) clade, *ata* was found in 78% of the analyzed genomes. In contrast, within the clade representing *A. calcoaceticus, A. dijkshoorniae* and *A. pittii* (CDP clade), only 2% of the analyzed genomes contained *ata.*

The uneven distribution of *ata* across the analyzed species could indicate lineage-specific losses of this gene. Yet, lineage-specific differences in the quality of the genome assembly and the gene annotation could generate a similar pattern, since the repetitive structure of *ata* (Supp. Figure 1) complicates the genome assembly based on short read data. As a consequence, contig ends may often fall within the gene boundaries leaving the annotated gene incomplete or missed entirely by the annotation procedure. The length distribution of the encoded Ata proteins shows that this is indeed a common artefact (Supp. Figure 2). However, we did not find that this preferentially affects any species. To rule out that *ata* has been missed entirely in the CDP clade, we additionally considered gene order information. In *A. baumannii* ATCC 19606, Ata (RefSeq Protein Accession WP_001045602.1) is flanked upstream by two genes encoding the 23S rRNA-methyltransferase (WP_000702193.1) and the Lon protease (WP_001292274.1), and downstream by two genes coding for the outer membrane protein assembly factor TpgA (equivalent to BamE: WP_001044114.1) and the 5-formyltetrahydrofolate cyclo-ligase (WP_001004364.1), respectively. We determined the phylogenetic profiles of the four flanking genes and integrated them with the profile of *ata* (Figure 1(b)). This revealed that the occurrence of *ata* is tightly linked to that of *tpgA*, which encodes a periplasmic protein that forms a complex with Ata and assists in the transport of Ata to the cell surface [28]. Notably, the order of the five genes is entirely conserved in 93% of the *A. baumannii* genomes that harbor the *ata* gene, and where the assembly contiguity allows the detection of this microsyntenic region (i.e. the *ata* gene is neither located two genes up- nor downstream to a contig end). In turn, when *ata* was missing, almost always also *tpgA* was absent, and the genes encoding the Lon Protease and the cyclo-ligase reside next to each other.

**Figure 2.**
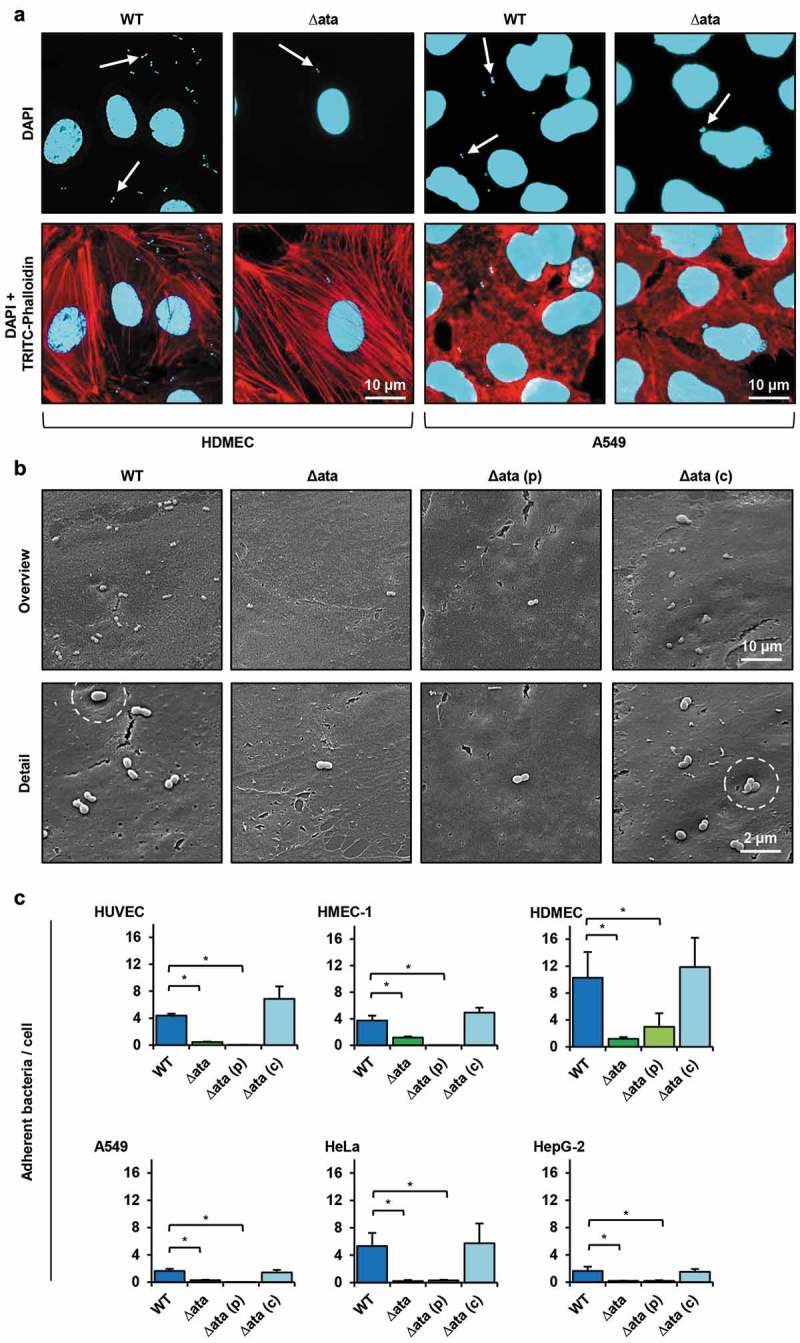
Ata-mediated adhesion to human endothelial or epithelial. (a) Representative fluorescence microscopy of infected endothelial and epithelial cells. Human cells (HDMEC or A549) were incubated with *A. baumannii* (MOI 200) for 1 h. Non-adherent bacteria were flushed and samples were fixed with paraformaldehyde. DNA was stained with DAPI (blue) and the cytoskeleton was stained with TRITC-phalloidin (red). Arrows indicate adherent bacteria. (b) Scanning electron microscopy of HUVEC-associated *A. baumannii*. HUVECs were incubated with *A. baumannii* (MOI 200) for 1 h. Non adherent bacteria were flushed, infected cells were fixed in 2.5% glutaraldehyde and prepared for scanning electron microscopy. (c) Ata-dependent adhesion of *A. baumannii* to human endothelial and epithelial host cells. Endothelial (HUVEC, HMEC-1, HDMEC) and epithelial cells (A549, HeLa, HepG-2) were incubated with *A. baumannii* (MOI 200) for 1 h. After incubation, planktonic bacteria were rinsed and infected cells were harvested by tryptic digestion. Samples were used for analyzing adherent bacteria by amplifying human and *A. baumannii* specific genes (*hmbs* and *rpoB*) in a qRT-PCR approach. C_T_-values, obtained from qRT-PCR, were used for calculation of bacterial adhesion. Values are means ± SEM of five independent experiments; *, p < 0.05.

We subsequently focused on *ata* in other *Acinetobacter* spp.. Similar to *A. baumannii*, the presence of *ata* typically implies the presence of *tpgA*, which presumably represents the ancestral state for *Acinetobacter*. Exemplarily for the 177 genomes of *A. pittii* harboring an Ata ortholog, we confirmed that the joint absence of *ata* and *tpgA* is most likely not an artefact of an inaccurate genome assembly. In 160 of these genomes, we find the two genes encoding the Lon protease and the 5-formyltetrahydrofolate cyclo-ligase residing next to each other on the same contig indicating a true loss of *ata* and *tpgA* (Supp. Figure 3). In the remaining 17 genomes, the Lon protease and 5-formyltetrahydrofolate cyclo-ligase encoding genes are disrupted by a contig break. Thus, we conclude that a joint loss of the functionally related gene pair, *ata*/*tpgA* explains the data best. In summary, the almost complete absence of *ata* in the CDP clade most likely represents a genuine evolutionary signal.

**Figure 3.**
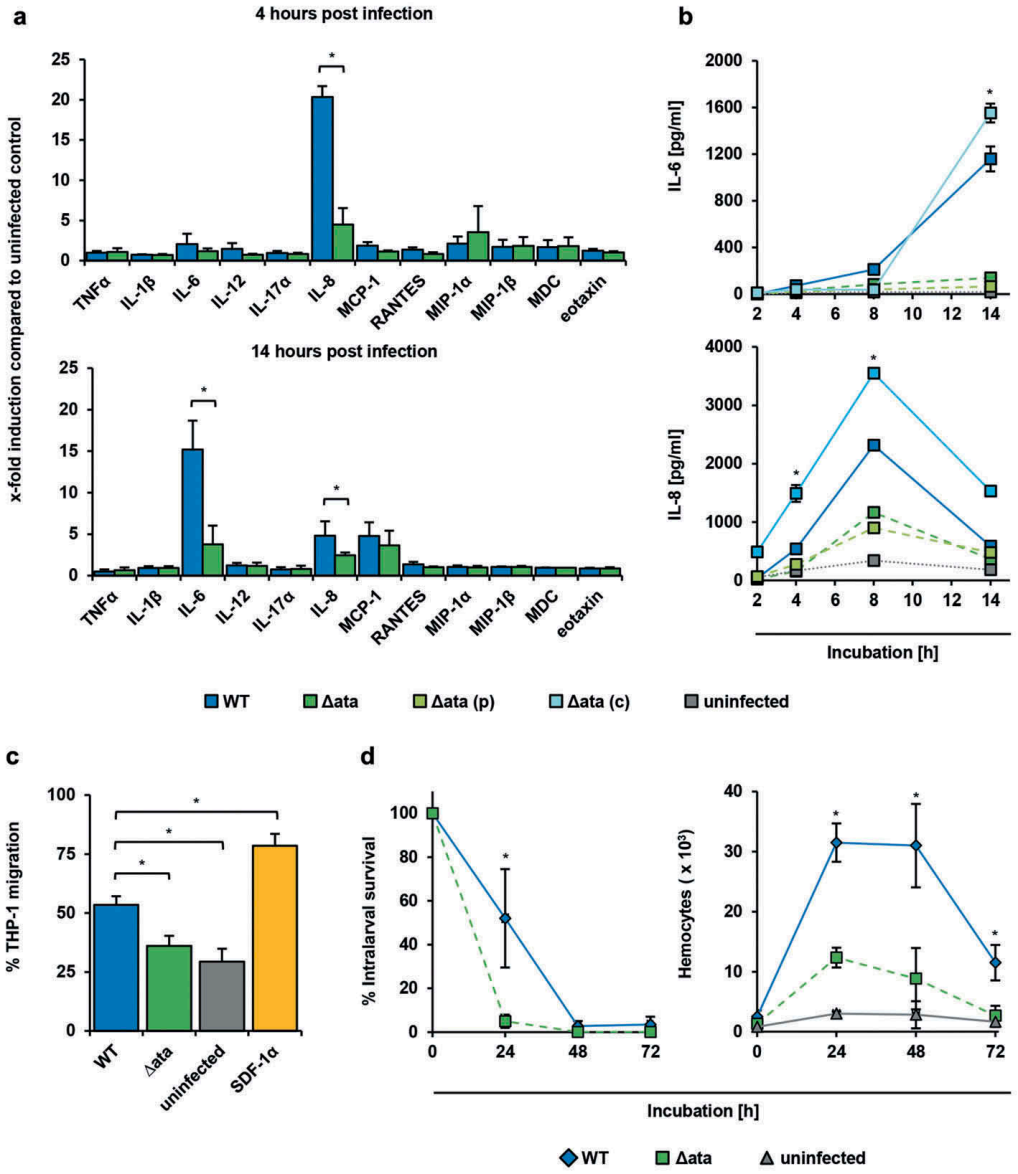
*A. baumannii* induce inflammatory response in endothelial cells and supports migration of immune cells. (a+b) Ata-mediated induction of inflammatory cytokines upon infection of HUVECs. HUVECs were incubated with *A. baumannii* (MOI 1) for the indicated time points, and levels of secreted chemokines and cytokines were determined in the supernatant by ELISA. Values are means ± SEM of six independent experiments; *, p < 0.05. (c) Infection of HUVECs with *A. baumannii* supports transmigration of THP-1 cells. Sterile filtered supernatants of *A. baumannii* infected HUVECs (MOI 1, 14 h) were used as chemoattractant for analyzing transmigration of THP-1 cells. Monocytes (5 × 10^5^) were placed into the upper part of a cell culture insert (pore size: 8 µm) and allowed to migrate for 16 h towards the chemoattractant in the lower part of the well. THP-1 cells were counted using trypan blue staining and a hemocytometer. (d) Survival of *A. baumannii* in *G. mellonella* and its contribution to activate hemocytes within the larvae. Caterpillars were infected with a sub-lethal dose of *A. baumannii* (1 × 10^5^ bacteria). For analyzing the survival of *A. baumannii*, larvae were homogenized at the indicated time points and serial dilutions were plated onto Endo agar (BD) for CFU enumeration. To investigate the activation of hemocytes, larvae were homogenized and centrifuged in a cell filter containing tube to separate the hemolymph. Samples were mixed with 100 µL of trypsin-EDTA (0.05%) and stained with trypan blue, immediately. Hemocytes were enumerated using a hemocytometer. In (c)+(d), values are means ± SD of three independent experiments; *, p < 0.05.

### Deletion and complementation of *ata* in *A. baumannii*

To analyze the function of Ata in *A. baumannii* we used the type strain ATCC 19606 and an isogenic, markerless deletion strain (∆*ata*) which had been generated previously [15]. In *A. baumannii* ATCC 19606, Ata consists of 2,258 amino acids with a predicted molecular mass of about 250 kDa in its monomeric form. For complementation purposes, ∆*ata* was transformed with the *ata* encoding expression vector pARKM_*ata* [22], thereby generating ∆*ata*_(c). Furthermore, ∆*ata* was transformed with the empty pARKM vector, thereby generating ∆*ata*_(p) which was used as an empty vector control. These four variants were used throughout this study: wild-type strain (WT), *ata* knock-out strain (∆*ata*), ∆*ata* complemented with pARKM_*ata* [∆*ata*_(c)] and ∆*ata* complemented with empty pARKM vector [∆*ata*_(p)].

Genomic or plasmid DNA was isolated from each strain and the presence or deletion of *ata* was verified by PCR and RFLP. A 6,777 bp fragment was amplified from WT DNA (Supp. Figure 4(a), lane 1), indicating the presence of *ata*, whereas no signal was observed when using DNA of ∆*ata* (lane 2). Restriction of pARKM from ∆*ata*_(p) and pARKM_*ata* from ∆*ata*_(c) with *Bam*HI/*Xba*I revealed the linearized vector (7,556 bp) (lane 5) or the linearized pARKM (7,556 bp) and a fragment of 6,777 bp (lane 7) respectively, indicating the successful transformations of *∆ata*.

**Figure 4.**
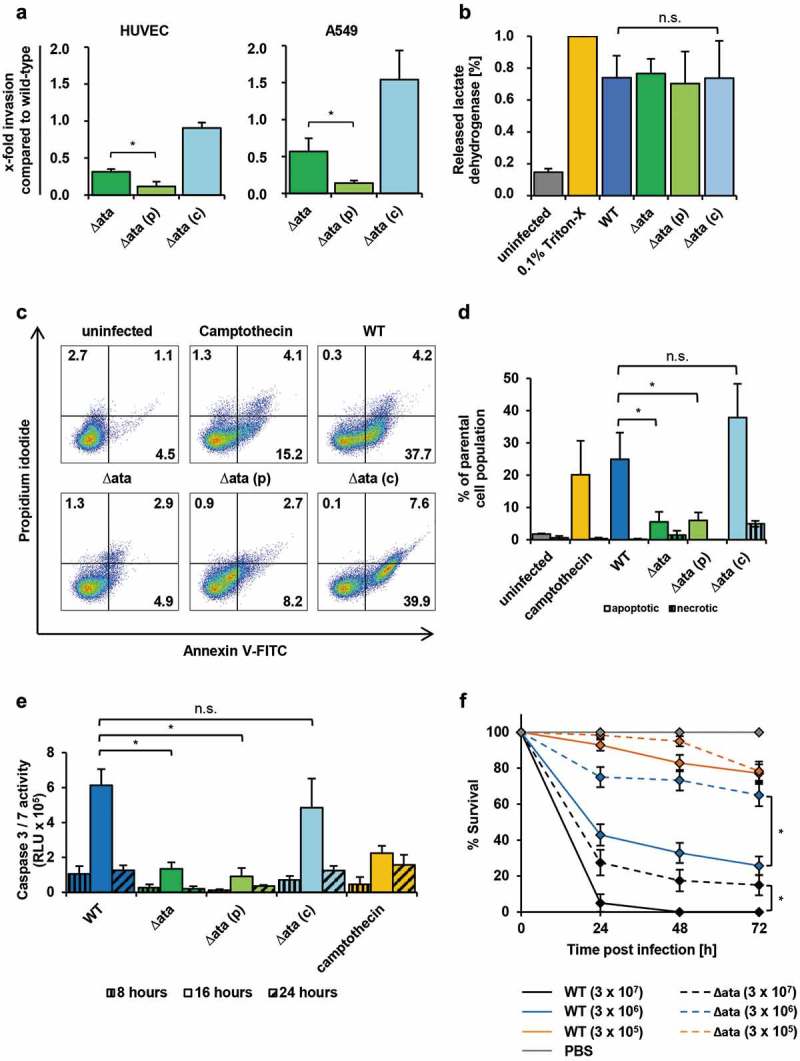
Role of Ata in mediating virulence in endothelial cells and *G. mellonella*. (a) Invasion of *A. baumannii* into HUVECs and A549 is Ata-mediated. Human cells were incubated with *A. baumannii* (MOI 200) for 4 h and non-bound bacteria were flushed. Extracellular bacteria were killed with gentamicin (500 µg/mL) or colistin (10 µg/mL). Intracellular bacteria were released after lysis of human cells with deionized water and CFUs were determined by plating serial dilutions. Invasion of the WT strain was set as 1. Values are means ± SD of five independent experiments; *, p < 0.05. (b) Release of lactate dehydrogenase (LDH) upon infection of HUVECs. Endothelial cells were infected with *A. baumannii* (MOI 200) for 24 h. The supernatant was sterile filtered and the amount of released LDH was determined by spectrophotometry. Uninfected and 0.1% Triton X-100 treated cells were used as negative or positive control respectively. In (b)-(e), values are means ± SD of three independent experiments; *, p < 0.05. (c+d) Ata induces apoptosis in endothelial cells. HUVECs were infected with *A. baumannii* (MOI 1) for 16 h and stained with propidium iodide/Annexin V-FITC and analyzed by flow cytometry to determine viable, apoptotic and necrotic cells. Gates indicating different stages of cell death: lower left (viable), lower right (early apoptosis), upper right (late apoptosis) and upper left (necrosis). Numbers represent percentage of parental cell population. (e) *A. baumannii* upregulates caspase-3 and caspase-7. *A. baumannii* (MOI 1) were used for infection of HUVECs for the indicated time points. The activity of caspase-3 and caspase-7 was determined by fluorometry. Camptothecin (60 µM) was used as a positive control. (f) Ata-dependent lethality of *G. mellonella* larvae. Larvae were injected with the indicated CFUs of *A. baumannii* and the survival was monitored for 72 h. Values are means ± SEM of four independent experiments; *, p < 0.05.

For evaluation of *ata* gene expression, the respective *A. baumannii* strains were cultivated with (+) or without (-) arabinose and total RNA was analyzed by quantitative real-time PCR (qRT-PCR). A strong *ata* expression was observed in the WT strain (2.3 ± 0.4%) and in arabinose-induced ∆*ata*_(c) (3.3 ± 1.1%), whereas no signal was detected for ∆*ata* and ∆*ata*_(p) (Supp. Figure 4(b)). Low expression levels were found for ∆*ata*_(c) without arabinose (0.4 ± 0.2%), indicating a low background expression of *ata*, which is most likely due to the leakiness of the pBAD-promoter system [29]. To evaluate Ata production in WT, ∆*ata* and complemented strains, the surface localization was analyzed by immunofluorescence using an anti-Ata-head antibody. Fluorescence microscopy confirmed the surface location of Ata for WT and ∆*ata*_(c) strains, but not for ∆*ata* and ∆*ata*_(p) (Supp. Figure 4(c)).

Successful deletion and complementation of *ata* were confirmed by PCR, sequencing, RFLP, gene expression and immunofluorescence. Growth experiments in LB revealed no differences in doubling times between the single strains (data not shown).

### Adhesion of *A. baumannii* to endothelial and epithelial cells is ata-dependent

Since adhesion to host cells is important to establish infections, the Ata-dependent binding of *A. baumannii* to human cells was analyzed. For this purpose, primary endothelial cells (HUVEC and HDMEC) or endothelial (HMEC-1) and epithelial cell lines (A549, HeLa, HepG-2) were used. The interaction of *A. baumannii* with their respective host cells was analyzed by fluorescence and scanning electron microscopy. Ata-producing *A. baumannii* adhered more efficient to the host cells compared to *ata*-deficient bacteria for both, endothelial and epithelial cells (Figure 2(a+b)). Adhesion of WT bacteria to HDMECs was ~ 4-fold higher compared to A549 human lung epithelial cells as observed by fluorescence microscopy (Figure 2(a)). Scanning electron microscopy revealed a higher adhesion rate of WT and ∆*ata*_(c) on the surface of HUVECs compared to ∆*ata* and ∆*ata*_(p) (Figure 2(b)). Notably, *ata*-expressing *A. baumannii* were located in small pit-like structures on the surface of HUVECs, which could not been observed for the knock-out strains (Figure 2(b), white dashed circles).

Next, Ata-dependent adhesion of *A. baumannii* to human epithelial and endothelial cells was compared and numbers of adherent bacteria were quantified using a qRT-PCR approach [15]. For HUVECs, 4.4 ± 0.3 bacteria adhered per endothelial cell when infected with the WT strain (Figure 2(c)). This represents an approx. 10-fold higher adhesion compared to Δ*ata* (0.4 ± 0.05 bacteria/cell). When *ata* expression was induced in Δ*ata_*(c) by arabinose, the adhesion rate was more than 20-fold higher compared to Δ*ata_*(p) (6.9 ± 1.9 bacteria/cell and 0.03 ± 0.01 bacteria/cell respectively). Similar results were obtained when additional endothelial cell lines were employed: HMEC-1 (WT: 3.7 ± 0.8 bacteria/cell; ∆*ata*: 1.2 ± 0.2 bacteria/cell; Δ*ata_*(p): 0.01 ± 0.01 bacteria/cell; Δ*ata_*(c): 4.9 ± 0.8 bacteria/cell) or HDMEC (WT: 10.0 ± 3.9 bacteria/cell; ∆*ata*: 1.2 ± 0.3 bacteria/cell; Δ*ata_*(p): 3.0 ± 2.0 bacteria/cell; Δ*ata_*(c): 11.2 ± 4.4 bacteria/cell). Notably, when HDMECs were infected with *A. baumannii*, bacterial adhesion was twice as high compared to HUVECs and HMEC-1.

Deletion of *ata* almost completely abolished the ability of *A. baumannii* to bind to all three analyzed human epithelial cells, thereby confirming the results obtained with the endothelial cells. For A549 lung cells, 1.7 ± 0.3 bacteria/cell were detected when infected with the WT strain and 0.3 ± 0.07 bacteria/cell for ∆*ata*. When *ata* was induced in Δ*ata_*(c) by arabinose and compared with Δ*ata_*(p), the dependence of Ata for adhesion of *A. baumannii* was even more evident (Δ*ata_*(c): 1.4 ± 0.4 bacteria/cell vs. Δ*ata_*(p): 0.01 ± 0.01 bacteria/cell). Similar results were obtained when HepG-2 and HeLa cells were used.

The adhesion of *ata*-expressing *A. baumannii* to A549 and HepG-2 epithelial cells was considerably lower compared to all endothelial cells. Adhesion to HeLa cells was 3-fold higher than to A549 or HepG-2 cells and comparable to HUVEC and HMEC-1 but lower than for HDMEC, indicating that *A. baumannii* adheres at least as efficient to endothelial cells compared to epithelial cells. These results show that Ata is essential for adhesion of *A. baumannii* to human endothelial and epithelial host cells during infection.

### Ata induces IL-6 and IL-8 secretion in huvecs

Since *A. baumannii* interacted with endothelial cells in an Ata-dependent manner, we next analyzed the inflammatory response of infected HUVECs by quantification of secreted cytokines and chemokines using a multiarray ELISA approach. At 4 h post infection (p.i.), a 20-fold increase of IL-8 could be measured in supernatants of WT-infected HUVECs compared to the uninfected control (20.3 ± 1.4 x-fold), whereas secretion of IL-8 was significantly lower when HUVECs where infected with Δ*ata* (4.5 ± 2.1 x-fold) (Figure 3(a)). After 14 h, the amount of secreted IL-6 increased 15-fold compared to the uninfected control (15.2 ± 3.5 x-fold) and 4-fold compared to Δ*ata* (3.8 ± 2.3 x-fold), whereas the amount of IL-8 decreased (WT: 4.8 ± 1.7 x-fold; Δ*ata*: 2.7 ± 0.3 x-fold) (Figure 3(a)). The secretion of the macrophage attracting protein (MCP-1) was slightly induced, but this was independent of Ata (WT: 4.8 ± 1.7 x-fold; Δ*ata*: 3.7 ± 1.8 x-fold). To verify these findings, we investigated the kinetics of IL-6 and IL-8 secretion upon infection of HUVECs with *A. baumannii* (Figure 3(b)). IL-8 levels peaked after 8 h (WT: 2.3 ± 0.002 ng/mL and 1.2 ± 0.02 ng/mL for Δ*ata*) and decreased over time, whereas IL-6 continuously increased over time up to late infection stages at 14 h (WT: 1.2 ± 0.1 ng/mL; Δ*ata*: 0.14 ± 0.02 ng/mL). These results indicate that HUVECs secret IL-8 during early stages and IL-6 during later stages of infection with *A. baumannii* in an Ata-dependent manner.

### Ata-dependent recruitment of immune cells

We next sought to address if the Ata-dependent pro-inflammatory cytokine secretion of HUVECs would recruit monocytes, which are important immune cells contributing to antimicrobial functions in tissue infections [30]. After co-cultivation of HUVECs with *A. baumannii*, cell culture supernatants were sterile filtered and used as chemoattractants in transwell migration assays employing THP-1 monocytes. A background transmigration of 29% was observed when cells were incubated with just medium (“uninfected”), whereas 79% of THP-1 cells transmigrated when the chemokine SDF-1α, which has been described to activate migration of THP-1 cells [27], was used as a positive control. A transmigration rate of 53% was observed when supernatants of WT-infected HUVECs were used (Figure 3(c)). In contrast, transmigration was reduced to 36% when supernatants of Δ*ata*-infected HUVECs were applied.

To investigate the recruitment of immune cells *in vivo*, larvae of *Galleria mellonella* were injected with sublethal doses of 10^5^ *A. baumannii* and numbers of hemocytes in the hemolymph were quantified (Figure 3(d)). Hemocyte numbers increased 13-fold upon infection and dropped again after 48 h. Compared to Δ*ata* a 2.5-fold increase after 24 h of infection with the WT strain was observed, indicating that Ata has led to a higher inflammatory response in *G. mellonella* larvae. Hemocyte numbers inversely correlated with the number of surviving bacteria (Figure 3(d)), and loss of Ata correlated with a reduced survival of Δ*ata* within the larvae. At 24 h p.i. almost all Δ*ata* bacteria were killed, whereas ~ 55% of the WT bacteria were still viable (Figure 3(d)). After 48 h, both WT and ∆*ata* strain were killed.

### *A. baumannii* invades into epithelial and endothelial cells in an ata-dependent manner

*A. baumannii* has been described to invade human epithelial cells [6], and based on these findings, we investigated Ata dependence of invasion into endothelial and epithelial cells using a gentamicin/colistin protection assay. HUVECs or A549 cells were infected with *A. baumannii* for 4 h and treated with gentamicin or colistin to kill extracellular bacteria. Compared to the WT strain, a strong reduction in invaded cells could be observed for Δ*ata* (31 ± 3%) and Δ*ata_*(p) (12 ± 6%), whereas invasion by Δ*ata_*(c) (91 ± 7%) was not influenced (Figure 4(a)). Likewise, in A549 cells, the invasion rate for ∆*ata* and Δ*ata_*(p) decreased to 57 ± 18% and 14 ± 3% respectively, whereas invasion was not diminished when the Δ*ata_*(c) strains was employed.

### Ata induces apoptosis in huvecs

To evaluate the impact of Ata on cytotoxicity and induction of apoptosis, HUVECs were infected with the WT, the Δ*ata* strain and complemented controls. The release of the intracellular enzyme lactate dehydrogenase (LDH) upon infection was measured as a surrogate marker for cytotoxicity. After infection of HUVECs with the WT strain, LDH activity was determined to be 74 ± 14% and 77% ± 9% for Δ*ata*, indicating that Ata does not mediate membrane disintegration by *A. baumannii* (Figure 4(b)).

Apoptosis and necrosis of HUVECs was measured by propidium iodide/Annexin V staining and flow cytometry (Figure 4(c)). Upon infection with *A. baumannii* the highest level of apoptosis were induced after 16 h; the apoptosis rate after 12 h was <5% and cell death after 20 h consisted of mainly secondary necrotic cells (data not shown). Infection of HUVECs with *A. baumannii* WT at 16 h p.i. resulted in an apoptosis rate of 25 ± 8% of the parental cell population, whereas <2% of cells were necrotic (Figure 4(d)). When HUVECs were infected with ∆*ata*, only 5 ± 3% of the HUVECs became apoptotic. As apoptosis can be induced by caspase-dependent as well as caspase-independent mechanisms [31], we further assessed the activity of the executioner caspases, caspase-3 and caspase-7. WT infected HUVECs showed a 5-fold higher activity of caspase-3 and caspase-7 compared to ∆*ata* infected HUVECs with the highest levels between 8 h and 24 h (Figure 4(e)). These results indicate that infection of HUVECs by WT *A. baumannii* is associated with higher rates of caspase-dependent apoptosis, but not necrosis, in comparison to ∆*ata*.

### Ata mediates virulence *in vivo*

To analyze the impact of Ata on pathogenicity of *A. baumannii in vivo*, the *Galleria mellonella* larvae infection model was employed. Different CFUs were injected into the hemocoel of the larvae and the survival was monitored over a period of 72 h. To compare virulence between WT and Δ*ata* strains, time-kill curves were assessed and median lethal doses (LD_50_) were determined. Both strains caused a time- and dose-dependent killing of larvae (Figure 4(f)). After injection of 3 × 10^7^ bacteria nearly all infected *G. mellonella* died after 24 h p.i., whereas the survival rate increased to 40% and 90% when 3 × 10^6^ and 3 × 10^5^ bacteria were injected respectively. In contrast, after injection of *∆ata* approximately 30% of the larvae were still alive after 24 h. Mortality of WT infected larvae was always higher for each time point when comparing to larvae infected with the Δ*ata* strain. Likewise, LD_50_ values were lower for the WT at each time point, indicating that loss of Ata attenuates virulence of *A. baumannii in vivo* (**Supplement Table 2**). Notably, when an *ata* transposon mutant of *A. baumannii* ATCC 17978 was compared with its parental strain in the *Galleria* infection model, the impact of Ata for killing of larvae was even higher **(Supplement Table 2)**.

### Discussion

We investigated the impact of the trimeric autotransporter adhesin Ata of *A. baumannii* on infection of human host cells and *G. mellonella* larvae. We found that Ata acts as a multifunctional virulence factor of *A. baumannii* by (I) mediating adhesion to and invasion into human endothelial and epithelial cells, (II) inducing secretion of the pro-inflammatory cytokines IL-6 and IL-8 in HUVECs, (III) inducing apoptosis of HUVECs in a caspase-dependent manner and (IV) contributing to virulence *in vivo*. These results strongly suggest that Ata serves as an important virulence factor of *A. baumannii* during infections in human and insect models.

TAAs are multifunctional proteins and important virulence factors in Gram-negative bacteria [32]. For example, the expression of *yadA* from *Y. enterocolitica* strongly correlates with virulence, adhesion and host cell modulation [33–37]. BadA of *B. henselae* induces the hypoxia inducible factor-1 to promote angiogenesis and mediates adhesion to ECMs and endothelial cells [15,38–40]. Several studies showed that various *A. baumannii* strains attach to epithelial cells [16], and it is strongly believed that this cell type represents the main target of *A. baumannii* in host tissues. However, recent studies indicate that *A. baumannii* also interacts with endothelial cells as they are associated with blood stream or soft tissue infections [15,41,42]. We indeed found that *A. baumannii* adheres to endothelial and epithelial cells in an Ata-dependent manner (Figure 2(a+b)). Adhesion of *A. baumannii* to endothelial cells occurred on average at higher frequencies compared to epithelial cells (Figure 2(c)).

Former studies demonstrated that *A. baumannii* is capable of invading human epithelial cells *in vitro* [6,17]. We demonstrated that the deletion of *ata* significantly diminished invasion of *A. baumannii* into HUVECs and A549 cells. This might be, at least partially, due to the strong Ata-dependent adhesion defect, since the number of intracellular bacteria depends on the ability of bacteria to attach to human host cells. TAA-mediated invasion into host cells is not uncommon. For example, YadA of *Y. pseudotuberculosis* mediates the uptake of the bacteria into human cells by a fibronectin-dependent bridging between the TAA and β_1_-integrins [43]. *A. baumannii* expressing *ata* were frequently localized in pits on the surface of infected HUVECs (Figure 2(b)), which might represent an early stage in the invasion process.

Bacterial virulence factors can stimulate the host response by triggering the secretion of inflammatory cytokines or chemokines. We observed that *A. baumannii* induce IL-6 and IL-8 in an Ata-dependent mode and activates monocyte transmigration (Figure 3(a–c)). In line with our findings, previous studies showed that *A. baumannii* induce inflammation by stimulation of TNF-α, IL-6 and IL-8 in epithelial cells and the IL-8 homolog keratinocyte-derived chemoattractant in mice [44,45]. A modest induction of MIP-1α in HUVECs after 4 h p.i. and a stronger induction of MCP-1 after 14 h p.i. were observed for both the WT and Δ*ata*, which likely have contributed to transmigration (Figure 3(a+d)). However, even though we analyzed the secretion of 12 important chemokines by HUVECs upon infection with *A. baumannii* we cannot rule out that other chemokines might be involved [46,47].

In this context, it could be demonstrated that secreted outer membrane vesicles (OMV) of *A. baumannii* activate the inflammation in host cells [48]. It can be assumed that huge immunodominant outer membrane proteins like Ata directly interact with the host tissue and thereby stimulate diverse inflammatory responses. In fact, also other TAAs such as BadA or YadA can trigger the production of cytokines or chemokines, e.g. the vascular endothelial growth factor or IL-8 [10,37]. Compared to the Ata induced inflammatory response, YadA stimulates the IL-8 secretion at earlier stages of infections with a maximum for IL-8 after 6 h [37].

To analyze the contribution of Ata-mediated *A. baumannii* pathogenicity *in vivo*, we employed the *G. mellonella* infection model, which has been shown to be suitable for analyzing virulence of *A. baumannii* and the innate immune response [49–52]. Infection of larvae with the ∆*ata* and the WT strains revealed that deletion of *ata* led to significantly impaired time-kill kinetics clearly indicating that Ata mediates pathogenicity *in vivo* (Figure 4(f)). Sublethal doses of *A. baumannii* were then injected and intralarval survival of the bacteria was monitored. Survival of *ata*-expressing *A. baumannii* was higher in *G. mellonella* after 24 h of incubation in comparison to *ata* deletion strains (Figure 3(d)). In line with the Ata-dependent pro-inflammatory human cell response, the number of larval hemocytes (which are similar to phagocytes in humans [53]) increased, indicating that *A. baumannii* might activate the recruitment of larval hemocytes by Ata (Figure 3D). The higher intralarval survival of the WT compared to Δ*ata* despite the increased recruitment of phagocytic cells could, at least partially, be explained if Ata would somehow mediate protection against the host. This has indeed been extensively described for the autotransporter YadA from *Y. enterocolitica* and BadA from *Bartonella henselae* which e.g. mediate immune evasion, serum resistance and antiphagocytosis [10,12,33–37,43]. However, if this is also applicable to Ata needs to be addressed in further studies.

Ata mediated apoptosis of HUVECs at 12–20 h p.i., but did not influence cytotoxicity or necrosis (Figure 4(c+d)). During bacterial infections, apoptosis can be either induced by the extrinsic pathway (via death receptors) or intrinsic pathway (via mitochondria) [54]. For *A. baumannii* both mechanisms could be demonstrated [55–57]. *A. baumannii* activates the effector caspase-3 and Poly (ADP-ribose) polymerase (PARP) [57]. We observed that Ata also stimulates the cleavage of caspase-3 and in addition caspase-7 in HUVECs (Figure 4(e)). Even though most bacteria inhibit apoptosis to preserve their replicative niche, the destruction of endothelial or epithelial barriers by the induction of apoptosis might be beneficial for *A. baumannii* to enter deeper tissues.

We have elucidated the role of Ata during the infection of human tissue and have shed initial light on the underlying molecular mechanisms based on *A. baumannii* ATCC 19606 as a showcase. Using the phylogenetic profiles of *ata*, and of its flanking genes in *A. baumannii* ATCC 19606 across 3,052 *Acinetobacter* spp. allowed us to embed these findings into a functional evolutionary context. Ata orthologs represent the full phylogenetic diversity of the genus *Acinetobacter*. Most likely Ata was already present before the contemporary *Acinetobacter* spp. started diversifying. We find the genes encoding Ata and its functionally interacting partner, TpgA, in the majority of *A. baumannii* strains, and also in most genomes of the closely related species *A. nosocomialis* and *A. seifertii*. Thus, the three species share the same fundamental machinery to facilitate cell adhesion. Accordingly, we propose that the findings exemplarily made for *A. baumannii* ATCC 19606 describe a mode of infection that is representative for the entire NSB clade. It is tempting to speculate that the loss of Ata in the NSB clade correlates with a reduced virulence. However, the situation is more complex. *A. baumannii* AYE is an epidemic strain with a 26% mortality rate in infected individuals [58]. Yet, this strain lacks both *ata* and *tpgA*, and this can neither be explained with the quality of the genome assembly nor with that of the gene annotation. This suggests that, similar to other virulence factors (e.g. *bauA, omp33-36* and *pglC* [59]), alternative factors exist that can maintain virulence even when *ata* is absent. The almost complete deletion of the *ata*/*tpgA* gene pair in the CDP clade should be interpreted with caution. Individual studies exist, which propose that members of this clade have an attenuated virulence, which might at least be partially due to absence of *ata* [60,61]. Yet, a decisive conclusion must await a thorough description of facultative virulence factors that can replace Ata, and a more precise determination if, and to what extent, virulence is indeed attenuated in the CDP clade.

In summary, we show that Ata trigger multiple important steps for the initiation of successful infections in host cells. To elucidate the key functions of *A. baumannii* infection, further research should focus on the host-pathogen interface by e.g. deciphering human host cell receptors and mechanism of apoptosis or immune evasion.
