# Supplementary material for: The Acinetobacter trimeric autotransporter adhesin Ata controls key virulence traits of Acinetobacter baumannii
Source: Virulence. 2019 Jan 14;10(1):68–81. doi: 10.1080/21505594.2018.1558693 (PMC6363060; doi:10.1080/21505594.2018.1558693)
Supplement: Supplemental Material [file kvir-10-01-1558693-s001.zip › Supplement_Figure 1_.pptx]

## Slide 1
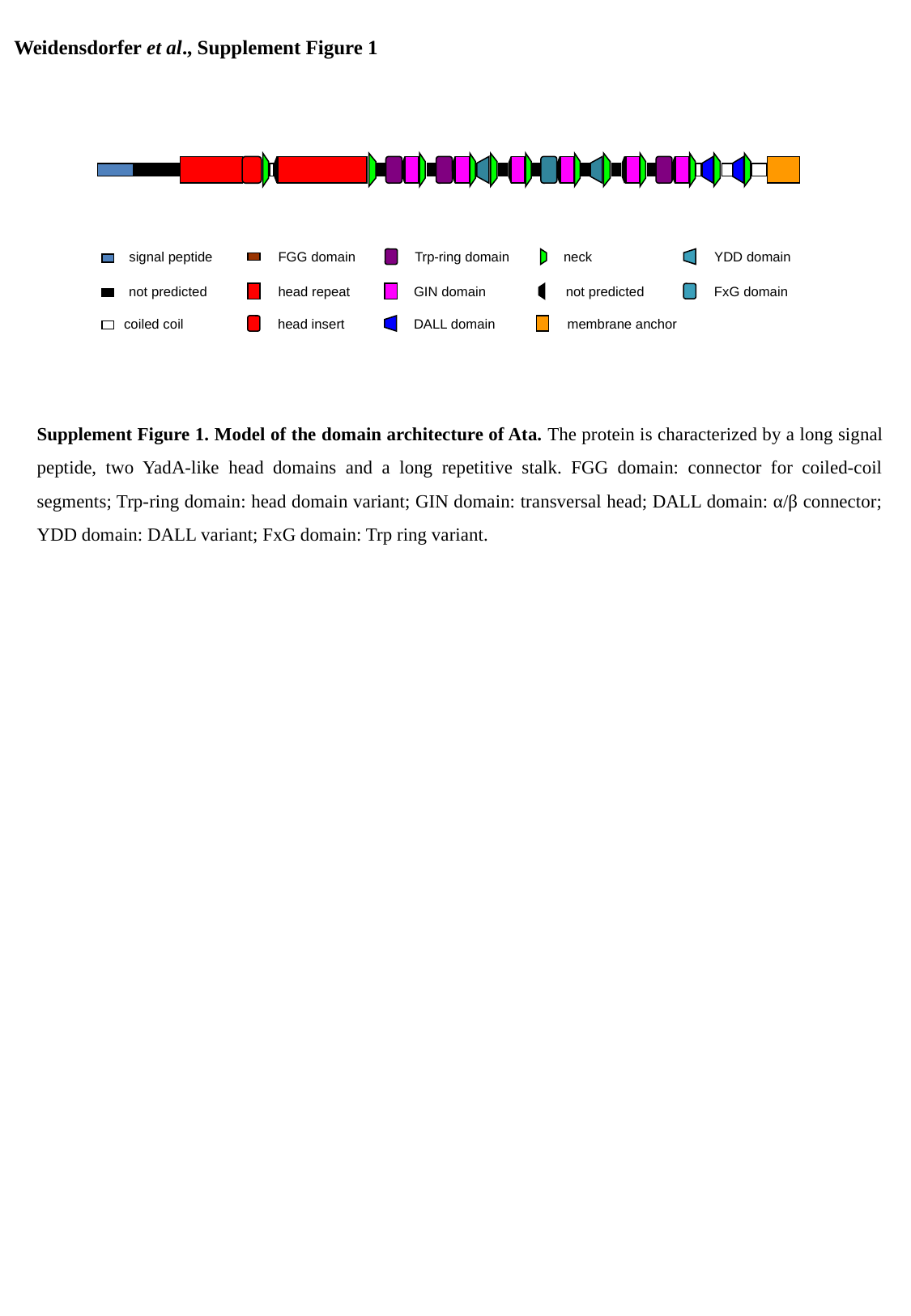

Weidensdorfer et al., Supplement Figure 1
signal peptide
FGG domain
Trp-ring domain
neck
YDD domain
not predicted
head repeat
GIN domain
not predicted
FxG domain
coiled coil
head insert
DALL domain
membrane anchor
Supplement Figure 1. Model of the domain architecture of Ata. The protein is characterized by a long signal peptide, two YadA-like head domains and a long repetitive stalk. FGG domain: connector for coiled-coil segments; Trp-ring domain: head domain variant; GIN domain: transversal head; DALL domain: α/β connector; YDD domain: DALL variant; FxG domain: Trp ring variant.
