# Supplementary material for: The Acinetobacter trimeric autotransporter adhesin Ata controls key virulence traits of Acinetobacter baumannii
Source: Virulence. 2019 Jan 14;10(1):68–81. doi: 10.1080/21505594.2018.1558693 (PMC6363060; doi:10.1080/21505594.2018.1558693)
Supplement: Supplemental Material [file kvir-10-01-1558693-s001.zip › Supplement_Figure 2.pptx]

## Slide 1
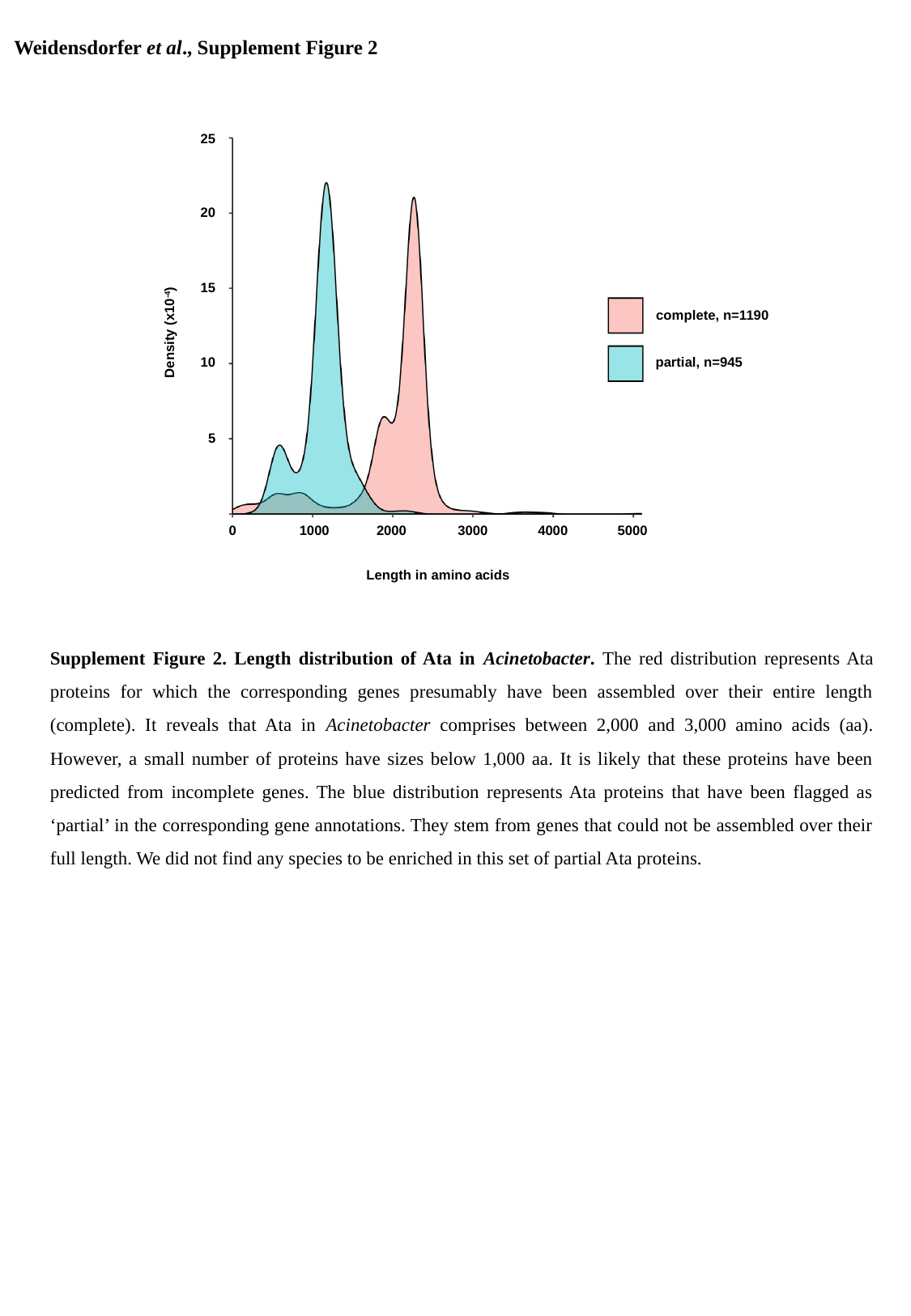

Weidensdorfer et al., Supplement Figure 2
25
20
15
complete, n=1190
Density (x10-4)
10
partial, n=945
5
0
1000
2000
3000
4000
5000
Length in amino acids
Supplement Figure 2. Length distribution of Ata in Acinetobacter. The red distribution represents Ata proteins for which the corresponding genes presumably have been assembled over their entire length (complete). It reveals that Ata in Acinetobacter comprises between 2,000 and 3,000 amino acids (aa). However, a small number of proteins have sizes below 1,000 aa. It is likely that these proteins have been predicted from incomplete genes. The blue distribution represents Ata proteins that have been flagged as ‘partial’ in the corresponding gene annotations. They stem from genes that could not be assembled over their full length. We did not find any species to be enriched in this set of partial Ata proteins.
