# Supplementary material for: The Acinetobacter trimeric autotransporter adhesin Ata controls key virulence traits of Acinetobacter baumannii
Source: Virulence. 2019 Jan 14;10(1):68–81. doi: 10.1080/21505594.2018.1558693 (PMC6363060; doi:10.1080/21505594.2018.1558693)
Supplement: Supplemental Material [file kvir-10-01-1558693-s001.zip › Supplement_Figure 3.pptx]

## Slide 1
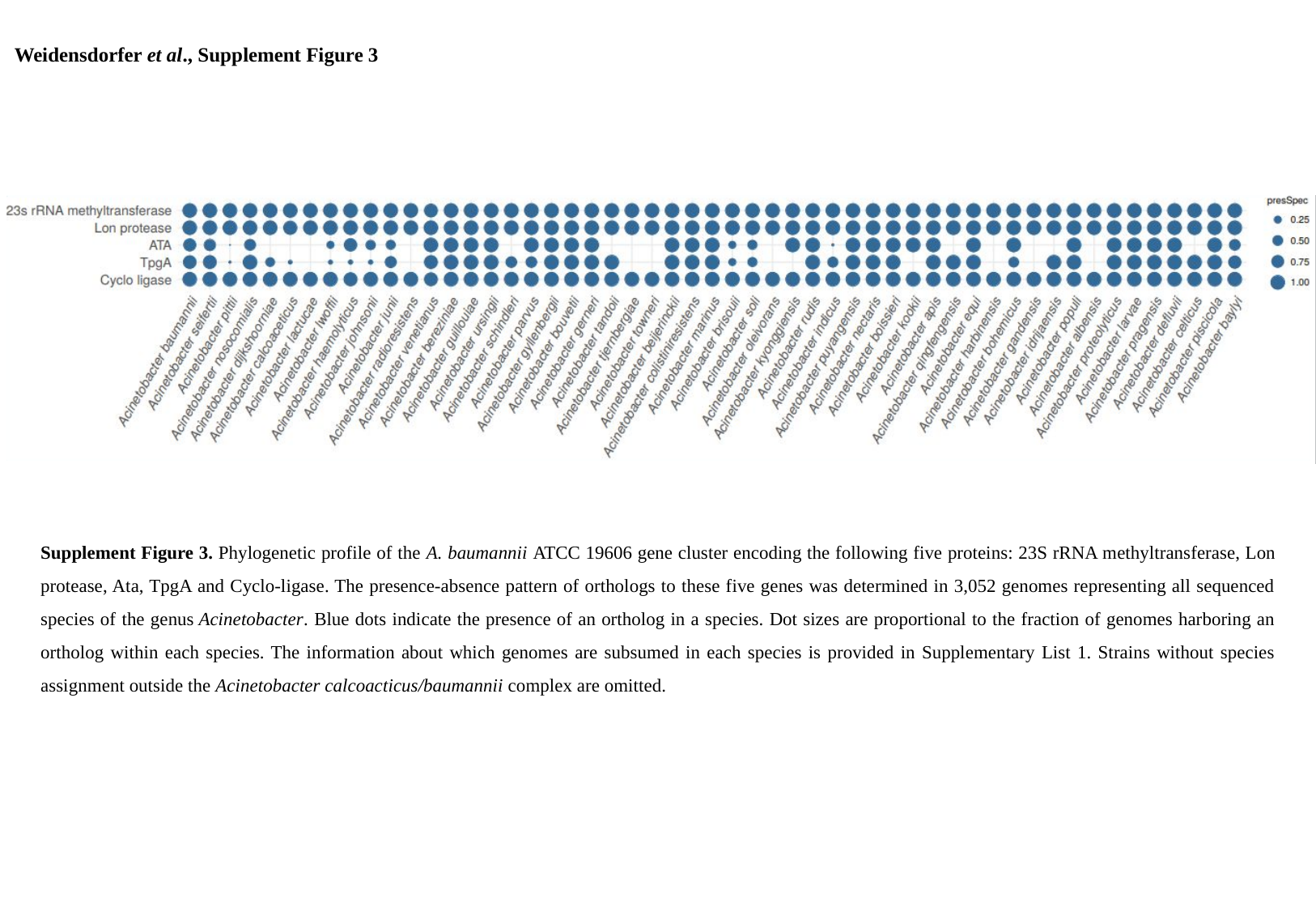

Weidensdorfer et al., Supplement Figure 3
Supplement Figure 3. Phylogenetic profile of the A. baumannii ATCC 19606 gene cluster encoding the following five proteins: 23S rRNA methyltransferase, Lon protease, Ata, TpgA and Cyclo-ligase. The presence-absence pattern of orthologs to these five genes was determined in 3,052 genomes representing all sequenced species of the genus Acinetobacter. Blue dots indicate the presence of an ortholog in a species. Dot sizes are proportional to the fraction of genomes harboring an ortholog within each species. The information about which genomes are subsumed in each species is provided in Supplementary List 1. Strains without species assignment outside the Acinetobacter calcoacticus/baumannii complex are omitted.
