# Supplementary material for: The Acinetobacter trimeric autotransporter adhesin Ata controls key virulence traits of Acinetobacter baumannii
Source: Virulence. 2019 Jan 14;10(1):68–81. doi: 10.1080/21505594.2018.1558693 (PMC6363060; doi:10.1080/21505594.2018.1558693)
Supplement: Supplemental Material [file kvir-10-01-1558693-s001.zip › Supplement_Figure 4.pptx]

## Slide 1
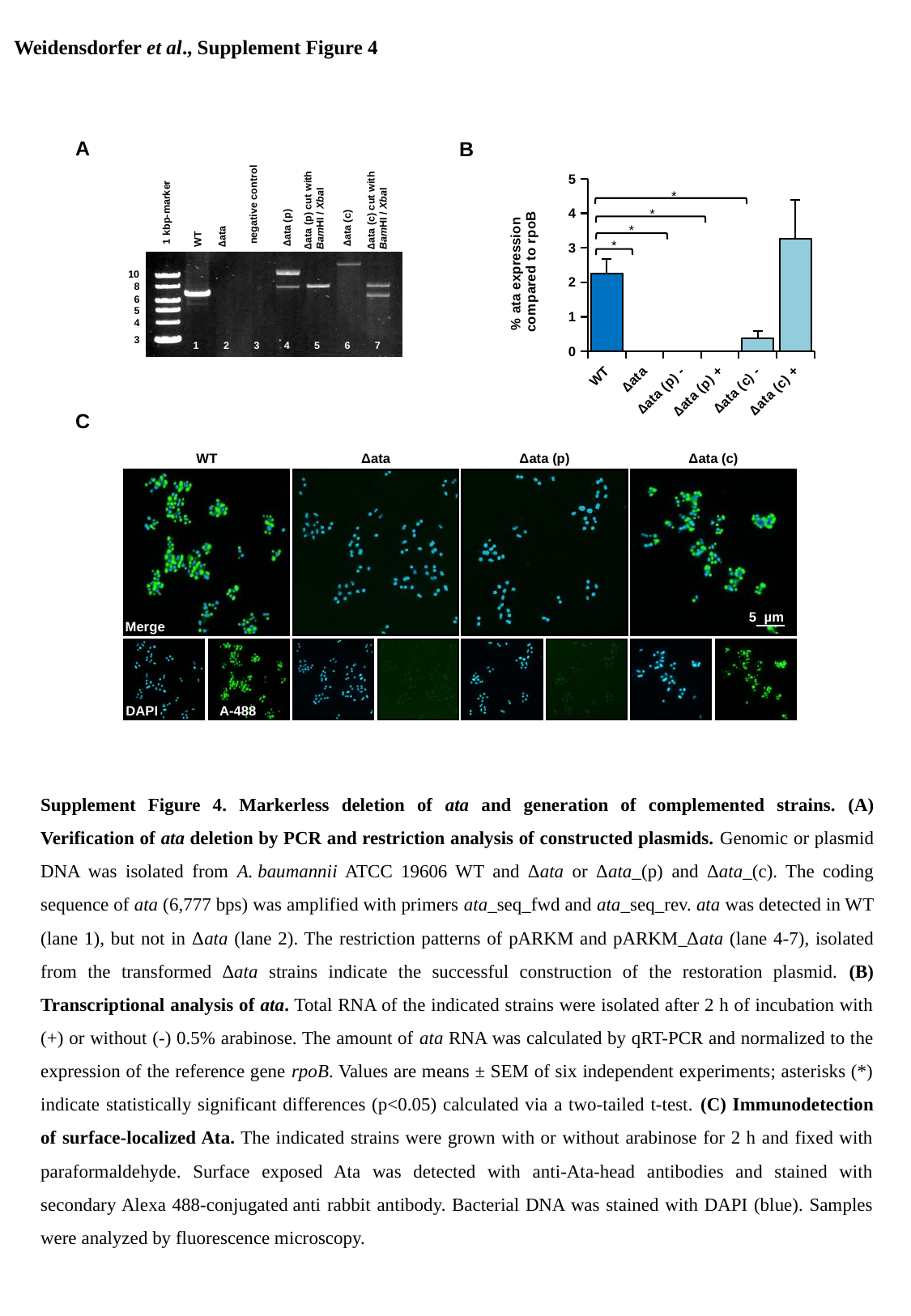

Weidensdorfer et al., Supplement Figure 4
∆ata (p) cut with
BamHI / XbaI
negative control
1 kbp-marker
∆ata (p)
∆ata (c)
∆ata
WT
10
8
6
5
4
3
∆ata (c) cut with
BamHI / XbaI
A
B
### Chart
| Category | |
|---|---|
| WT | 2.2555509001869822 |
| ∆ata | 0.0017441440969695897 |
| ∆ata (p) - | 0.009665144500145674 |
| ∆ata (p) + | 0.004228123408676143 |
| ∆ata (c) - | 0.37639073492978653 |
| ∆ata (c) + | 3.2675780721350622 |*
*
*
*
1
2
3
4
5
6
7
C
WT
Δata
Δata (p)
Δata (c)
5 µm
Merge
DAPI
A-488
Supplement Figure 4. Markerless deletion of ata and generation of complemented strains. (A) Verification of ata deletion by PCR and restriction analysis of constructed plasmids. Genomic or plasmid DNA was isolated from A. baumannii ATCC 19606 WT and Δata or Δata_(p) and Δata_(c). The coding sequence of ata (6,777 bps) was amplified with primers ata_seq_fwd and ata_seq_rev. ata was detected in WT (lane 1), but not in Δata (lane 2). The restriction patterns of pARKM and pARKM_∆ata (lane 4-7), isolated from the transformed ∆ata strains indicate the successful construction of the restoration plasmid. (B) Transcriptional analysis of ata. Total RNA of the indicated strains were isolated after 2 h of incubation with (+) or without (-) 0.5% arabinose. The amount of ata RNA was calculated by qRT-PCR and normalized to the expression of the reference gene rpoB. Values are means ± SEM of six independent experiments; asterisks (*) indicate statistically significant differences (p<0.05) calculated via a two-tailed t-test. (C) Immunodetection of surface-localized Ata. The indicated strains were grown with or without arabinose for 2 h and fixed with paraformaldehyde. Surface exposed Ata was detected with anti-Ata-head antibodies and stained with secondary Alexa 488-conjugated anti rabbit antibody. Bacterial DNA was stained with DAPI (blue). Samples were analyzed by fluorescence microscopy.
