# Supplementary material for: The Acinetobacter trimeric autotransporter adhesin Ata controls key virulence traits of Acinetobacter baumannii
Source: Virulence. 2019 Jan 14;10(1):68–81. doi: 10.1080/21505594.2018.1558693 (PMC6363060; doi:10.1080/21505594.2018.1558693)
Supplement: Supplemental Material [file kvir-10-01-1558693-s001.zip › Supplement_Figure 5.pptx]

## Slide 1
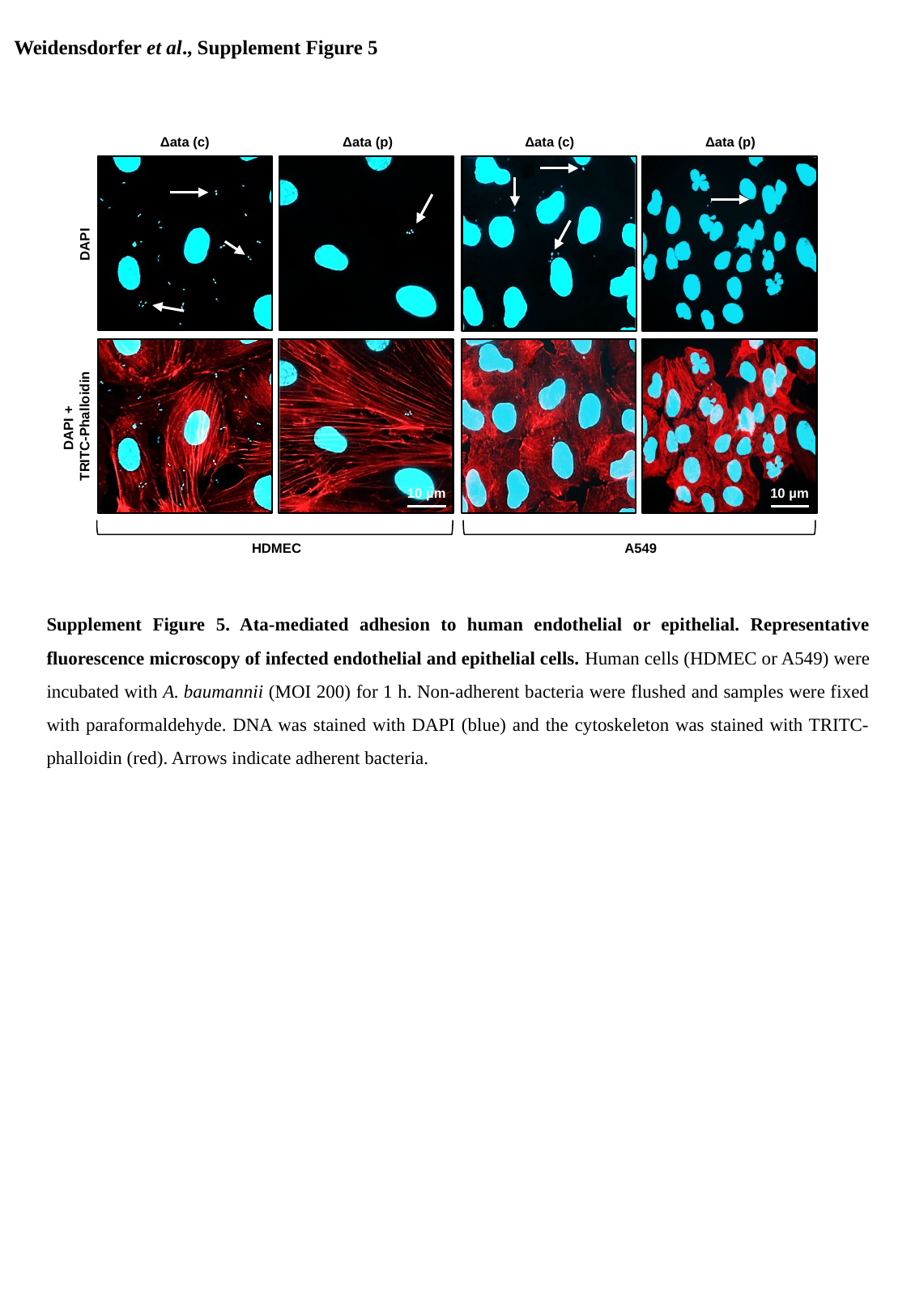

Weidensdorfer et al., Supplement Figure 5
Δata (c)
Δata (p)
Δata (c)
Δata (p)
DAPI
DAPI +
TRITC-Phalloidin
HDMEC
A549
10 µm
10 µm
Supplement Figure 5. Ata-mediated adhesion to human endothelial or epithelial. Representative fluorescence microscopy of infected endothelial and epithelial cells. Human cells (HDMEC or A549) were incubated with A. baumannii (MOI 200) for 1 h. Non-adherent bacteria were flushed and samples were fixed with paraformaldehyde. DNA was stained with DAPI (blue) and the cytoskeleton was stained with TRITC-phalloidin (red). Arrows indicate adherent bacteria.
