# Supplementary material for: The Acinetobacter trimeric autotransporter adhesin Ata controls key virulence traits of Acinetobacter baumannii
Source: Virulence. 2019 Jan 14;10(1):68–81. doi: 10.1080/21505594.2018.1558693 (PMC6363060; doi:10.1080/21505594.2018.1558693)
Supplement: Supplemental Material [file kvir-10-01-1558693-s001.zip › Weidensdorfer_Supplement Methods_.docx]

**Weidensdorfer *et al*.,** Ata-mediated virulence of *A. baumannii*

**Supplementary Methods**

***Generation of an anti-Ata-antibody***

The head-coding sequence (nucleotide position from 4 to 2,049, NCBI Reference Sequence: WP_001045627.1) of *ata* was amplified with *ata*_head_*Nde*I_fwd/ *ata*_head_*Xho*I_rev, digested with *Nde*I and *Xho*I and ligated into pET24a, resulting in pET24a_*ata*-head (**Table 1**). *E. coli* BL21 (DE3) was transformed with pET24a_*ata*-head (**Supp.** **Table 1**). Bacteria were grown until an OD_600_ of 1.0 and protein production was induced with 100 µM isopropyl β-D-1-thiogalactopyranoside (IPTG) for 3 h at 37 °C. Following induction, bacteria were harvested and lysed by sonication. The His-tagged Ata-head (~68 kDa, monomeric) was purified from crude extract by affinity-chromatography (Ni-NTA, Sigma-Aldrich) and used for immunization of rabbits by a commercial provider (Eurogentec). The isolated polyclonal anti-Ata-antibodies (rabbit IgG) were purified using the NAb Protein A/G Spin Kit (Thermo Fisher) according to the manufacturer's protocol.

***Cultivation of human cell lines and bacterial strains***

Primary human umbilical cord vein cells (HUVECs) were prepared from fresh cord veins according to the ethical permission (4/12; University Hospital Frankfurt am Main) as described [1]. They were further cultivated in endothelial growth medium supplemented with growth factor mix (Promocell) and 10% fetal calf serum (FCS, Sigma-Aldrich). Human microvascular endothelial cells (HMEC-1, CRL-3243, Promocell) and human dermal microvascular endothelial cells (HDMEC, C-12215, Promocell) were grown in microvascular endothelial growth medium supplemented with growth factor mix (Promocell) and 1% FCS. A549 human lung epithelial cells (ACC-107, DSMZ), human cervical epithelial cells (HeLa, ACC 57, DSMZ), human hepatic epithelial cells (HepG-2, ACC 180, DSMZ) and human monocytes (THP-1, ACC 16, DSMZ) were cultivated in RPMI medium (Biochrom) with 10% FCS. All human cells were cultivated in 75 cm² cell culture flasks in a humidified incubator with a 5% CO_2_ atmosphere at 37 °C.

Bacteria used in this study are listed in **Table 1**. They were cultivated in LB at 37 °C. For infection experiments, the OD_600_ of *A. baumannii* cultures were adjusted to 0.05 and bacteria were incubated to an OD_600_ of 0.2 (approx. 1x10^8^ CFU/mL). The transformants ∆*ata*(p) and ∆*ata*(c) were grown in LB containing 100 µg/mL of kanamycin and *ata* expression in ∆*ata*(c) was induced by the addition of 0.5% arabinose. *E. coli* strains DH5α, BL21-DE3 and WM6026 used for cloning purposes were cultivated in LB containing 30 µg/mL of kanamycin.

***RNA isolation and quantification of ata gene expression***

Bacteria were grown as described above and harvested by centrifugation at OD_600_ of 0.2. RNA isolation was done by chloroform-phenol-extraction using Max Bacterial Enhancement buffer and TRIzol (both Thermo Fisher) according to the manufacturer's protocol. The amount of *ata* or *rpoB* transcripts in 50 ng total RNA was analyzed by qRT-PCR using primer pairs *rpoB*-RT-fwd/*rpoB*-RT-rev and *ata*-RT-fwd/*ata*-RT-rev (**Supp.** **Table 1**). C_T_ values of the amplified fragments were determined as previously described [2]. Calculation of gene copy numbers was done employing pTOPO_*rpoB* or pTOPO_*ata* (**Table 1**) of known concentrations as an external calibrator.

***Immunostaining of Ata***

Bacteria were grown until an OD_600_ of 0.2 was reached, washed in PBS and spotted onto glass slides for fixation with 3.75% paraformaldehyde. Samples were washed and blocked with 5% normal goat serum (Thermo Fisher) in PBS + 0.1% Tween-20 (PBS-T) overnight at room temperature. Next, cells were washed and incubated with purified polyclonal anti-Ata-antibody (1:100 in PBS-T) for 1 h followed by incubation with secondary Alexa 488 conjugated anti-rabbit antibody (1:1000 in PBS-T, Cell Signaling) for another hour. Finally, bacterial DNA was stained with 4',6-diamidino-2-phenylindole (DAPI), mounted in Fluoroprep (bioMérieux) and analyzed by fluorescence microscopy.

**In vitro *cytotoxicity measuring LDH release of infected HUVECs***

HUVECs were grown in 6-well plates and infected with *A. baumannii* (MOI 200) for 30 h. As a positive control, cells were treated with 0.1% Triton X-100. After incubation, supernatants were filtered (pore size: 0.45 µm) and the activity of released lactate dehydrogenase (LDH) was determined by spectrophotometry (Cobas 8000, module 701, Roche).

***Quantification of cytokines by enzyme-linked immunosorbent assay (ELISA)***

HUVECs were grown in 6-well plates and infected with *A. baumannii* at a MOI of 1 for the indicated time points. After incubation, cells were scraped from the wells using a cell scraper and lysed by sonication. Cell debris were centrifuged and the supernatants were used for quantifying amounts of produced cytokines and chemokines by a multi-analyte ELISA (Microbial-induced Multi-Analyte ELISArray Kit, Qiagen) or by human IL-6 or IL-8 ELISA kit (BD Bioscience) according to the manufacturer’s instructions. Uninfected samples were used as negative controls.

***Transmigration of THP-1 to infected HUVECs***

Transmigration of THP-1 cells was evaluated as described [3]. Briefly, HUVECs were infected with *A. baumannii* (MOI 1) for 4 and 14 h. Supernatants of infected cells were filtered (pore size: 0.45 µm) and used as chemoattractant in a THP-1 cell transmigration assay. THP-1 cells (5x10^5^) were added to the upper compartment of transwell inserts (pore size: 8 µm, Greiner BioOne) in a 24-well plate and the supernatant of the infected HUVECs was filled into the lower compartment. Uninfected HUVECs or 100 ng/mL of SDF-1α (PrepoTech) were used as a negative and positive control. THP-1 cells were allowed to transmigrate for 16 h in a humidified chamber at 37 °C. The number of non-migrated THP-1 cells in the upper part of the transwell insert was determined by trypan blue staining and enumeration using a hemocytometer. The efficiency of transmigration is given as the percentage of migrated THP-1 after 16 h of incubation compared to initial cell amount.
