# Supplementary material for: The Acinetobacter trimeric autotransporter adhesin Ata controls key virulence traits of Acinetobacter baumannii
Source: Virulence. 2019 Jan 14;10(1):68–81. doi: 10.1080/21505594.2018.1558693 (PMC6363060; doi:10.1080/21505594.2018.1558693)
Supplement: Supplemental Material [file kvir-10-01-1558693-s001.zip › Weidensdorfer_Supplement Tables_.docx]

**Weidensdorfer *et al.*,** Ata-mediated virulence of *A. baumannii*

**Supplementary Tables**

**Supplementary Table 1. Oligonucleotides**

| **Primer** | **Sequence (5’→3’)** | **Reference** |
| --- | --- | --- |
| *rpoB*-RT-fwd | GAG TCT AAT GGC GGT GGT TC | [4] |
| *rpoB*-RT-rev | ATT GCT TCA TCT GCT GGT TG | [4] |
| *hmbs*-RT-fwd | TTC CTT CCC TGA AGG GAT TCA CTC AG | [5] |
| *hmbs*-RT-rev | TTA AGC CCA GCA GCC TAT CTG ACA CCC | [5] |
| *ata*-RT-fwd | ATT CGG TGC TGT TGC ACA AG | this study |
| *ata*-RT-rev | CAC CCG GTT TAT TAC CAG AG | this study |
| Kan_*Pvu*II_fwd | CAG CAG CTG TTG ATC TTT TCT ACG GGG TCT G | this study |
| Kan_*Pvu*II_rev | CAG CAG CTG CTT AGA AAA ACT CAT CGA GCA TC | this study |
| *ata*-in_fusion-fwd | TTG GGC TAG CGA ATT CGT ATT TGT CTG AGA AGT TTT AT | this study |
| *ata*-in_fusion-rev | TGG CGG CCG CTC TAG ATT AAT TAA TCA CAC CAC TAA TAC | this study |
| *ata*_head_*Nde*I_fwd | ATG CAT ATG AAT AAA GTT TAC AAG GTC ATT TGG | this study |
| *ata*_head_*XhoI*_rev | ACC TTG GCC CTC GAG AAC TGC AAC TG | this study |
| *ata*_seq_fwd | GTC GTT GAG TTC GGT ATT TGT CTG AGA AG | this study |
| *ata*_seq_rev | TAA TCT CTT TAA TCT GTC GAG CAA GGA GAG | this study |

*underlined characters indicate restriction sites used for cloning

**Supplementary Table 2. Median Lethal Doses (LD_50_) of *Acinetobacter baumannii* Strains in Larvae of *Galleria mellonella* at 24 h, 48 h and 72 h p.i.**

|  | 24 h | 48 h | 72 h |
| --- | --- | --- | --- |
| ***A. baumannii* strain** | **LD_50_ (x 10^6^ CFUs)**  72 h | | |
| ATCC 19606 WT | 2.56 ± 0.06 | 1.80 ± 0.06 | 1.33 ± 0.07 |
| ATCC 19606 ∆*ata* | 4.02 ± 0.11 | 3.16 ± 0.11 | 2.40 ± 0.09 |
|  | **LD_50_ (x 10^5^ CFUs)** | | |
| ATCC 17978 WT | 0.52 ± 0.01 | 0.17 ± 0.01 | 0.10 ± 0.01 |
| ATCC 17978 ∆*ata* | 2.15 ± 0.14 | 1.45 ± 0.15 | 0.86 ± 0.16 |

References

[1] Baudin B, Bruneel A, Bosselut N, *et al*. A protocol for isolation and culture of human umbilical vein endothelial cells. Nat Protoc. 2007;2:481–485.

[2] Whelan JA, Russell NB, Whelan MA. A method for the absolute quantification of cDNA using real-time PCR. Journal of Immunological Methods. 2003;278:261–269.

[3] Zaitseva L, Murray MY, Shafat MS, *et al*. Ibrutinib inhibits SDF1/CXCR4 mediated migration in AML. Oncotarget. 2014;5:9930–9938.

[4] Hornsey M, Ellington MJ, Doumith M, *et al*. AdeABC-mediated efflux and tigecycline MICs for epidemic clones of *Acinetobacter* *baumannii*. J Antimicrob Chemother. 2010;65:1589–1593.

[5] Weidensdorfer M, Chae JI, Makobe C, *et al*. Analysis of endothelial adherence of *Bartonella* *henselae* and *Acinetobacter* *baumannii* using a dynamic human *ex vivo* infection model. Infect. Immun. 2016;84:711–722.
